# Sales and regulatory status of fixed dose combination psychotropic drugs in India: a retrospective longitudinal study

**DOI:** 10.1080/20523211.2024.2372089

**Published:** 2024-08-06

**Authors:** Paul Bogowicz, Aashna Mehta, Shruti Choudhary, Petra Brhlikova, Peter Roderick, Patricia McGettigan, Habib Hasan Farooqui, Aditya Narain Sharma, Allyson M. Pollock

**Affiliations:** a Population Health Sciences Institute, Newcastle University, Newcastle upon Tyne, UK; b Indian Institute of Public Health – Delhi, Public Health Foundation of India, Gurugram, India; c William Harvey Research Institute, Queen Mary University of London, London, UK; d College of Medicine, Qatar University, Doha, Qatar; e Translational and Clinical Research Institute, Newcastle University, Newcastle upon Tyne, UK

**Keywords:** Fixed dose combination, psychiatry, psychotropic, psychopharmacology, pharmacoepidemiology, India

## Abstract

**Background:**

There is limited evidence to support use of fixed dose combination (FDC) drugs in the treatment of psychiatric disorders. This study aimed to examine the sales and regulatory status of psychotropic FDCs in India, in the context of two government regulatory initiatives.

**Methods:**

Official documents were searched to establish an account of the initiatives and measures targeting psychotropic FDCs. This was integrated with private market data (2008 to 2020). Descriptive statistics were used to examine changes in FDC numbers/formulations and sales volumes in standard units (SU) over time.

**Results:**

Psychotropic FDC sales volumes (percentage market share) increased from 0.8 billion SU (18.4%) in 2008 to 1.4 billion SU (20.1%) in 2020. The numbers (formulations) of FDCs also increased, from 28 (101) in 2008 to 33 (143) in 2020. Unapproved FDCs accounted for 69.3% of psychotropic FDC sales in 2008, decreasing slightly to 60.3% in 2020. Of 21 psychotropic FDCs considered under the regulatory initiatives, three went on to be banned, and two of these remained on the market in 2020.

**Conclusions:**

Unapproved FDCs continue to account for most psychotropic FDC sales, potentially putting the public at risk because their safety and efficacy have not been evaluated.

## 1. Introduction

**Figure 1.**
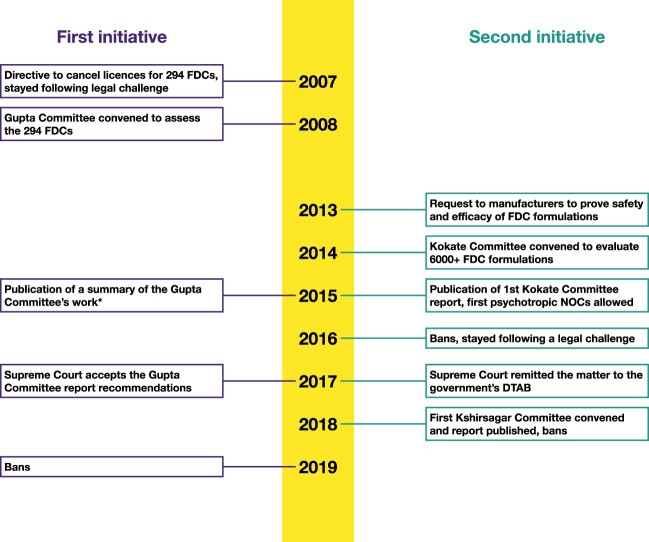
Key events relating to government initiatives to regulate unapproved FDCs. * The Gupta Committee report could not be located. DTAB = Drugs Technical Advisory Board, FDC = fixed dose combination, NOC = no objection certificate.

Fixed dose combinations (FDCs) contain two or more drugs in a single pharmaceutical form, such as a capsule. Many FDCs available in India lack approval from the central regulator, the Central Drug Standards Control Organisation (CDSCO) (Vendoti, 2018), meaning that their safety and efficacy have never been evaluated. A detailed analysis of India's drugs regulations found that central approval has been required for new drugs since 1961 (McGettigan et al., 2015). While CDSCO has overall responsibility for assessing the safety and efficacy of drugs, state regulators have significant responsibilities, including issuing manufacturing licences to pharmaceutical companies. In some cases, these licences have been issued for FDCs that lack central approval (Sharma, 2021). There have been concerns about the sale of such unapproved FDCs since the 1970s (Drugs Consultative Committee, 1978). The Indian government launched two initiatives to regulate the sale of unapproved FDCs, instead of considering criminal proceedings as recommended by a former senior Ministry of Health and Family Welfare official (Sharma, 2021). The first was a 2007 directive to state regulators to cancel manufacturing licences for 294 FDCs. It is not known why these particular FDCs were selected from the many unapproved FDCs that were marketed at the time. The second was a 2013 request by CDSCO to state regulators to ask pharmaceutical companies to ‘prove the safety and efficacy’ of FDC formulations (specific strength and pharmaceutical form) which had been licensed by states without CDSCO's prior approval (Drugs Controller General (India), 2013). Both initiatives faced legal challenges, brought by the pharmaceutical industry, culminating in Supreme Court rulings in 2017. The initiatives ultimately led to regulatory measures, such as ‘no objection [to sales] certificates’ (NOCs), where manufacturers of drugs deemed to be ‘rational’ could apply for de facto approval without formal regulatory assessment of safety and efficacy. A chronology of key events may be found in Figure 1.

There is limited evidence supporting the use of FDCs in the treatment of psychiatric disorders. A systematic review looking at studies published up until 2013 found nine randomised controlled trials: seven for olanzapine + fluoxetine and two for amitriptyline + chlordiazepoxide (Farooq & Singh, 2015). The review also found an earlier generation of studies but these were excluded because of methodological problems, such as use of diagnostic criteria of uncertain validity and use of impressions of improvement rather than standardised measures. There are no explicit recommendations regarding use of FDCs in publicly available Indian clinical guidelines for treating psychiatric disorders and no such FDCs in the National List of Essential Medicines (Avasthi & Grover, 2018; Avasthi et al., 2019; Gautam et al., 2018, 2019, 2017a, 2017b; Grover & Avasthi, 2019a, 2019b; Grover et al., 2017; Gupta et al., 2017; Janardhan Reddy et al., 2017; Ministry of Health & Family Welfare, 2015; Praharaj et al., 2018; Shah et al., 2017; Subramanyam et al., 2018).

Few studies have examined the sale or use of psychotropic FDCs (FDCs intended for the treatment of psychiatric disorders) in India. One study found that FDCs accounted for a third of private oral antipsychotic drug sales in 2011–12, of which 43% were for unapproved formulations (McGettigan et al., 2015). Such data are highly relevant as private expenditure on drugs constituted 69.4% of total pharmaceutical expenditure in India in 2017–18 and there is limited provision of drugs in public settings (High Level Expert Group on Universal Health Coverage, 2011; National Health Accounts Technical Secretariat, 2021).

It is unclear whether the 2007 (‘first’) and 2013 (‘second’) regulatory initiatives have affected the market for unapproved psychotropic FDCs. The initiatives and resultant measures appear to have had a limited impact on the sale of other types of FDCs, such as those containing antibiotics (Brhlikova et al., 2023). This study aimed to examine the sales and regulatory status of psychotropic FDCs in India over time, in the context of initiatives taken by the government to control unapproved FDCs, and with reference to regulation in major international markets such as the European Union (EU) and the United States (US).

## 2. Methods

### 2.1. Design

This was a retrospective longitudinal study, combining pharmaceutical sales data and data from regulatory documents. The focus was on FDCs appearing in the sales data.

### 2.2. Setting and data sources

#### 2.2.1. Sales data

The source of the sales data was PharmaTrac, a commercial database of Indian pharmaceutical sales compiled by AIOCD Pharmasofttech AWACS Pvt Ltd (AIOCD Pharmasofttech AWACS Pvt Ltd., 2011). PharmaTrac aggregates data from thousands of stockists from across India, which are then extrapolated to estimate national-level sales. Variables include sales volumes, as numbers of packs, and sales values. Pack sizes were used to calculate sales in standard units (SU), the numbers of capsules or tablets sold for each formulation. The data cover from 2008 to 2020. PharmaTrac was chosen over other databases due to pre-existing access to and familiarity with this data. Government databases are not as readily accessible, as each state has its own procurement processes, and are not comprehensive.

FDCs listed in PharmaTrac were screened by PBo (who has expertise in psychiatry) for those containing psychotropic drugs. For the purposes of this study, FDCs were regarded as psychotropic if they contained at least one psychotropic drug and were clearly marketed for psychiatric indications. The indication was determined on the basis of the categorisation within PharmaTrac (relevant groups under the ‘NEURO/CNS’ supergroup), approved indication, regulatory documents, online pharmacy websites, and through discussion with PM (who has expertise in clinical pharmacology) and the rest of the research team. Those marketed for non-psychiatric indications only, such as neuropathic pain, were excluded.

#### 2.2.2. Regulatory status

Indian regulatory approval status was determined by reading CDSCO's new drug approvals lists and by searching the approved drugs online database (Central Drugs Standard Control Organization, 2022a, 2022c). The EU, United Kingdom (UK), and US regulatory approval statuses were ascertained from searching the relevant databases (European Medicines Agency, 2022; Medicines & Healthcare Products Regulatory Agency, 2022; US Food & Drug Administration, 2022).

#### 2.2.3. Regulatory initiatives

The recommendations from the Gupta Committee were extracted from annexures to meeting minutes summarising its report, obtained via Right to Information request (Drugs Technical Advisory Board, 2018); the report could not be located. Recommendations from the Kokate and Kshirsagar Committees were extracted from their reports (Kokate Committee, 2015a, 2015b, 2016a, 2016b; Kshirsagar Committee, 2018). The Gupta and Kokate Committees categorised FDCs and FDC formulations, respectively, using labels such as ‘rational’ and ‘irrational’; while the general meaning of the terms used is clear, neither Committee provided definitions of the terms or guidance explaining how the categorisation was operationalised.

#### 2.2.4. Regulatory measures

Documents issued by CDSCO were searched for NOC (de facto approval) status (Drugs Controller General (India), 2020, 2021b). The banning status was determined by reading CDSCO's banned drugs list (Central Drugs Standard Control Organization, 2022b) and cross-referencing with the corresponding Gazette of India notifications (Ministry of Health & Family Welfare, n.d.). Legal judgements were reviewed by PR (who has expertise in law) for two FDCs, flupentixol + melitracen and imipramine + diazepam (see Supplemental Material – Appendices A and B).

### 2.3. Measures

The primary outcome measure was monthly sales volumes in SU at the level of each FDC formulation. Formulations were regarded as distinct if they had a different specified strength or pharmaceutical form, as per the Kokate Committee. For example, paroxetine + clonazepam 25 mg/0.5 mg capsule, 25 mg/0.5 mg tablet, 25 mg/0.5 mg tablet controlled release, and 12.5 mg/0.5 mg tablet controlled release were regarded as four different formulations. The data were grouped according to psychotropic class: antipsychotic, antidepressant, and benzodiazepine/sedative. There were no FDCs containing mood stabilisers (apart from excluded combinations of gabapentinoids and antidepressants indicated for neuropathic pain) or stimulants in the PharmaTrac database.

FDCs containing drugs from two or more psychotropic classes were categorised using a hierarchy: antipsychotic > antidepressant > benzodiazepine/sedative. For example, an FDC such as sertraline + alprazolam, which contains an antidepressant (sertraline) and a benzodiazepine (alprazolam), would be categorised as an antidepressant FDC for the purposes of this study. The hierarchy was based on one used in a previous study (McGettigan et al., 2015).

Some FDC formulations listed within PharmaTrac were regarded as indeterminate in relation to regulatory status and/or committee recommendations. For formulation-specific status (NOCs and formal approvals) and committee recommendations (Kokate Committee), formulations were regarded as indeterminate if at least one of drug name, strength, or form was missing. For FDC-specific status (bans) and committee recommendations (Gupta and Kshirsagar Committees), formulations were regarded as indeterminate if drug name was missing. For example, escitalopram + clonazepam tablet with missing strength was regarded as indeterminate for NOC and formal approval status but not for ban status.

### 2.4. Analysis

Descriptive statistics were used to examine changes in sales over time. Key statistics included annual sales volumes for each FDC and FDC formulation and annual sales volumes as a proportion of the sum of sales of psychotropic FDCs and single drugs (market share). The number of FDCs was determined by calculating the sum of all unique combinations of two or more drugs, ignoring order. The number of FDC formulations was determined by calculating the sum of all specific strength and pharmaceutical form combinations appearing in PharmaTrac. Statistics were stratified according to variables of interest, such as approval status. Figures were produced using Keynote (Version 12.0; Apple Inc; United States). Three key years of interest were selected: baseline years for the first and second initiatives (2008 and 2014, respectively) and the most recent year available (2020).

### 2.5. Ethics and governance

This study was observational and only used aggregated population level data, therefore ethical approval was not required. The datasets generated and/or analysed during the current study are not publicly available due to the terms of the licence agreement with AIOCD Pharmasofttech AWACS Pvt Ltd.

## 3. Results

### 3.1. The Psychotropic FDC market 2008–2020

There were 35 psychotropic FDCs listed in PharmaTrac that had measurable sales volumes for at least one year between 2008 and 2020. Of the 35 FDCs, 30 had fully specified drug name data (Supplemental Material – Appendix Table C1). Of the 30 FDCs, 13 were antipsychotics, 11 were antidepressants, and six were benzodiazepine/sedatives. One FDC comprised four drugs, three comprised three drugs, and 26 comprised two drugs. Of the 30 FDCs, there was evidence of regulatory approval for six in India, two in the US, one in the EU, and none in the UK.

The number of psychotropic FDCs (formulations) marketed increased from 28 (101) in 2008 to 33 (143) in 2020 (Table 1). Note that the number of FDCs on the market varied by year as FDCs were brought to and removed from the market. The share of the overall psychotropic drug market accounted for by FDCs increased from 18.4% to 20.1% over the same period, driven by increasing sales of antidepressant and benzodiazepine/sedative FDCs. The proportion of overall psychotropic FDC sales accounted for by unapproved formulations fluctuated, increasing from 69.3% in 2008 to 75.5% in 2014, and then decreasing to 60.3% in 2020 (Table 2). The proportion of antipsychotic FDC sales accounted for by unapproved formulations increased from 49.7% in 2008 to 57.7% in 2020. Nearly all benzodiazepine/sedative FDC sales were for unapproved formulations across the study period.

**Table 1.** Psychotropic FDC numbers, formulations, sales volumes, and proportions between 2008 and 2020.

Category	2008	2009	2010	2011	2012	2013	2014	2015	2016	2017	2018	2019	2020
Antipsychotic													
FDCs	12	13	13	12	12	13	14	14	14	15	15	14	13
FDC formulations	40	39	40	39	36	36	48	50	51	53	57	52	50
Sales (millions SU)	287.4	327.5	382.4	394.6	414.6	399.9	358.5	291.3	304.4	310.4	360.1	373.3	401.9
Sales (as % of antipsychotic^†^)	32.9	35.0	36.6	37.0	37.6	34.0	30.3	23.6	24.2	23.8	26.2	27.2	27.5
Antidepressant													
FDCs	10	10	10	11	11	12	12	12	12	12	12	12	12
FDC formulations	41	44	48	49	52	56	63	62	65	64	62	65	65
Sales (millions SU)	410.4	463.9	491.9	513.8	552.0	618.7	685.7	744.0	762.9	773.2	818.7	793.8	834.3
Sales (as % of antidepressant^†^)	28.8	29.6	29.8	29.5	30.9	31.6	32.4	32.5	30.1	30.0	30.5	29.7	31.7
Benzodiazepine/sedative													
FDCs	5	6	6	7	7	7	7	7	7	7	7	7	7
FDC formulations	19	20	21	24	26	25	25	26	23	24	26	28	27
Sales (millions SU)	113.8	118.3	117.2	118.5	138.8	162.4	174.1	186.8	207.0	209.1	213.9	221.2	202.8
Sales (as % of benzo*/sedative^†^)	5.4	5.3	4.9	4.7	5.5	6.1	6.5	6.6	7.0	7.0	7.3	7.8	6.6
Total													
FDCs	28	30	30	31	31	33	34	34	34	35	35	34	33
FDC formulations	101	104	110	114	115	119	138	139	140	142	146	146	143
Sales (millions SU)	815.1	913.4	995.9	1032.1	1109.9	1186.3	1222.5	1225.8	1278.0	1296.5	1396.5	1392.0	1442.3
Sales (as % of psychotropic^†^)	18.4	19.2	19.5	19.5	20.5	20.5	20.4	19.2	19.0	18.9	20.0	20.2	20.1

* Benzodiazepine. FDC = fixed dose combination, SU = standard units.^†^ The denominator was the sum of psychotropic FDC and single drug sales volumes for each category and overall (i.e. in total). The number of FDCs was the sum of all unique combinations of two or more drugs, ignoring order. The number of FDC formulations was the sum of all specific strength and pharmaceutical form combinations. There were also a small number of FDC formulations for which the category was unclear, accounting for 0.2–0.5% of psychotropic FDC sales in each year.

**Table 2.** Unapproved psychotropic FDC numbers, formulations, sales volumes, and proportions between 2008 and 2020.

Category	2008	2009	2010	2011	2012	2013	2014	2015	2016	2017	2018	2019	2020
Antipsychotic													
FDCs	10	11	11	11	10	10	10	11	11	12	12	12	11
FDC formulations	26	25	26	26	24	22	26	28	30	32	31	28	26
Sales (millions SU)	142.7	147.6	164.8	162.7	162.2	188.7	311.5	239.2	248.0	252.6	245.2	225.8	232.0
Sales (as % of antipsychotic FDC^†^)	49.7	45.1	43.1	41.2	39.1	47.2	86.9	82.1	81.5	81.4	68.1	60.5	57.7
Antidepressant													
FDCs	8	8	8	9	9	10	10	10	10	10	10	10	10
FDC formulations	23	24	27	28	28	32	36	37	40	40	38	39	39
Sales (millions SU)	308.9	342.0	349.8	351.8	372.5	403.2	437.6	469.0	441.4	414.1	440.8	415.4	435.1
Sales (as % of antidepressant FDC^†^)	75.3	73.7	71.1	68.5	67.5	65.2	63.8	63.0	57.9	53.6	53.8	52.3	52.2
Benzodiazepine/sedative													
FDCs	4	5	5	6	6	6	6	6	6	6	6	6	6
FDC formulations	12	13	13	17	19	19	19	19	19	19	19	19	19
Sales (millions SU)	113.5	118.1	116.9	118.1	138.6	162.3	174.0	186.6	206.9	208.0	212.9	220.7	202.5
Sales (as % of benzo*/sedative FDC^†^)	99.8	99.9	99.7	99.7	99.8	99.9	99.9	99.9	99.9	99.5	99.5	99.8	99.8
Total													
FDCs	22	24	24	26	25	26	26	27	27	28	28	28	27
FDC formulations	61	62	66	71	71	73	81	84	89	91	88	86	84
Sales (millions SU)	565.1	607.7	631.5	632.6	673.3	754.2	923.2	894.8	896.3	874.7	899.0	862.0	869.7
Sales (as % of total FDC^†^)	69.3	66.5	63.4	61.3	60.7	63.6	75.5	73.0	70.1	67.5	64.4	61.9	60.3

* Benzodiazepine. FDC = fixed dose combination, SU = standard units.^†^ The denominator was psychotropic FDC sales volumes for each category and overall (i.e. in total). The number of FDCs was the sum of all unique combinations of two or more drugs, ignoring order. The number of FDC formulations was the sum of all specific strength and pharmaceutical form combinations.

### 3.2. Regulatory initiatives

#### 3.2.1. First initiative: Gupta Committee

When the Gupta Committee was convened in 2008, there were 28 psychotropic FDCs on the market available in 101 formulations. Of these, ten formulations of five FDCs had been formally approved by CDSCO (Supplemental Material – Appendix Table C2), accounting for 29.1% of the psychotropic FDC market in that year. The remainder of the market consisted of 61 unapproved formulations, accounting for 69.3% of psychotropic FDC sales, and 30 formulations of indeterminate approval status. Three of the 61 unapproved formulations were already banned, accounting for 0.1% of unapproved psychotropic FDC sales.

The Committee considered three FDCs, available in ten formulations, all of which were unapproved (Supplemental Material – Appendix Table C3). All contained a combination of a benzodiazepine with either another sedative or a beta-blocker. These accounted for 20.0% of unapproved psychotropic FDC sales and 13.9% of overall psychotropic FDC sales in 2008. All three FDCs were categorised as requiring further trial data. The Gupta Committee also considered nine FDCs containing psychotropic drugs for which there was no PharmaTrac sales data (Supplemental Material – Appendix Table C4).

#### 3.2.2. Second initiative: Kokate Committee

By the time the Kokate Committee was established in 2014, there were 34 psychotropic FDCs on the market available in 138 formulations. Of these, 11 formulations of five FDCs had been formally approved by CDSCO, accounting for 22.1% of the psychotropic FDC market in that year (two formulations of paroxetine + clonazepam were added and one formulation of flupentixol + melitracen was removed, Supplemental Material – Appendix Table C2). The remainder of the market consisted of 81 unapproved formulations, accounting for 75.5% of psychotropic FDC sales, and 46 formulations of indeterminate approval status. Three of the 81 unapproved formulations were already banned, accounting for less than 0.1% of unapproved psychotropic FDC sales.

The Committee considered 49 formulations of 21 FDCs, accounting for 87.4% of the psychotropic FDC market in 2014 (Supplemental Material – Appendix Table C5). Most were either combinations of an antidepressant and a benzodiazepine, a benzodiazepine/sedative and a beta-blocker, or an antipsychotic and trihexyphenidyl. Six formulations already had CDSCO approval, with the remaining 43 accounting for 96.0% of unapproved psychotropic FDC sales in 2014. Six formulations had already been considered under the first initiative; the Kokate Committee evaluated only one of them. Of the 44 formulations which were evaluated, nine (five FDCs) were categorised as irrational, seven (two FDCs) as rational, and 28 (12 FDCs) as requiring further data (Supplemental Material – Appendix Table C5). The Kokate Committee also considered nine FDC formulations containing psychotropic drugs for which the PharmaTrac sales data were either incomplete or absent (Supplemental Material – Appendix Table C6).

#### 3.2.3. Responses to evaluations of the Gupta and Kokate Committees

##### 3.2.3.1 NOCs and formal approvals

Of the seven formulations evaluated by the Kokate Committee as rational, six went on to be allowed de facto approval (NOC) in 2015 and 2016 (Supplemental Material – Appendix Table C7). The remaining formulation had been approved in 2004 and appears to have been submitted to the Committee in error. No formal approvals were given after 2010.

##### 3.2.3.2 Bans

In 2016, the government banned three FDCs corresponding to five of the nine formulations categorised by the Kokate Committee as irrational. The bans were challenged in court. The ban on imipramine + diazepam was annulled (see Supplemental Material – Appendix B). The three FDCs were reconsidered by the Kshirsagar Committee in 2018 and were recommended to be banned. Of these, only chlorpromazine + trihexyphenidyl and flupentixol + escitalopram went on to be banned (in September 2018). Flupentixol + melitracen, categorised as irrational by the Kokate Committee, was banned by the government in 2014 in a separate measure specific to that FDC. The ban was quashed in 2017 (see Supplemental Material – Appendix A). A further FDC considered by the Kokate and Kshirsagar Committees, trifluoperazine + imipramine + chlordiazepoxide + trihexyphenidyl, which was listed in PharmaTrac but with missing strength data, was also banned in 2018. Overall, three FDCs were banned as a result of the second initiative and no FDCs were banned as a result of the first.

##### 3.2.3.3 Requiring further data

It appears that phase IV trials were initially required for FDC formulations categorised by the Kokate Committee as requiring further data (Drugs Controller General (India), 2016a, 2016b, 2017a, 2017b). The industry successfully lobbied against this, and after CDSCO consulted with the Kokate Committee, it recommended in May 2019 that most FDC formulations did not require phase IV trials (Drugs Controller General (India), 2019). Similarly, a document specifying what data would be required for FDCs considered by the Gupta Committee was published in 2021 (Drugs Controller General (India), 2021a). The documents show that only three of the 14 psychotropic FDCs categorised as requiring further data under the initiatives were required to have phase IV trial data, with most required to have ‘active post-marketing surveillance’ instead (Supplemental Material – Appendix Tables C5 and C8).^1^

##### 3.2.3.4 Evidence of further responses

There was no evidence of any governmental or CDSCO response, such as a ban, for the three remaining formulations categorised by the Kokate Committee as irrational (three of etizolam + propranolol).

### 3.3. Sales in 2020 by regulatory status

**Figure 2.**
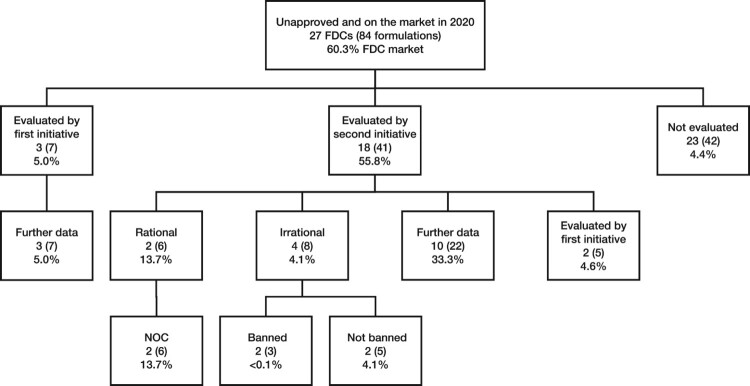
Unapproved FDCs and FDC formulations on the market in 2020, by outcome under the first and second government initiatives and regulatory status. FDC = fixed dose combination, NOC = no objection certificate. Each box contains FDC and FDC formulation numbers, as well as the proportion of the psychotropic FDC market accounted for by those formulations. Note that figures for sales of unapproved formulations include those for which CDSCO have allowed NOCs. All proportions use total psychotropic FDC sales in 2020 as denominator. Six FDC formulations were considered under both initiatives; this is reflected in the sum of formulations ‘Evaluated by first initiative,’ ‘Evaluated by second initiative,’ and ‘Not evaluated’ (7+41+42−6=84) as well as the corresponding market share (5.0%+55.8%+4.4%−4.9%=60.3%). The sum of the numbers of FDCs is much greater than 27 because of the overlap between initiatives and overlap between FDCs (in different formulations) ‘Evaluated by second initiative’ and ‘Not evaluated.’

Of the 33 psychotropic FDCs (143 formulations) on the market in 2020, 27 (84) lacked CDSCO approval, accounting for 60.3% of psychotropic FDC sales (Figure 2). Eight formulations categorised as irrational by the Kokate Committee remained on the market, accounting for 4.1% of FDC sales. Of the eight, three were banned, though these had very small sales volumes (<0.1%). No subsequent regulatory action was identified for the remaining five formulations. Many unapproved FDCs/FDC formulations were not assessed under either initiative, accounting for 4.4% of psychotropic FDC sales in 2020 (Figure 2). Most FDCs/FDC formulations that were not assessed were on the market by 2014, and therefore appear to have escaped assessment (30/42 formulations accounting for 3.8% of psychotropic FDC sales in 2020). Of the ten FDCs with the highest sales volumes in 2020, six had no evidence of CDSCO approval (Supplemental Material – Appendix Table C9). Most were combinations of a benzodiazepine/sedative and a beta-blocker or an antidepressant and a benzodiazepine/sedative.

### 3.4. Summary of the outcome of the regulatory initiatives

A summary of the results may be found in Box 1.

Box 1: Summary of findings.

The psychotropic FDC market 2008-2020

■ FDC numbers increased from 28 in 2008 to 33 in 2020 (17.9% increase)
■ FDC formulations increased from 101 in 2008 to 143 in 2020 (41.6% increase)
■ The proportion of the psychotropic market accounted for by FDCs increased from 18.4% in 2008 to 20.1% in 2020

Regulatory initiatives

■ First initiative (from 2007, Gupta Committee)
  ° Scope: considered 3 FDCs (10 formulations), accounting for 13.9% of psychotropic FDC sales at the time
  ° Recommendations: 3/3 requiring further data
  ° Regulatory change: none
■ Second initiative (from 2013, Kokate/Kshirsagar Committees)
  ° Scope: considered 49 formulations (21 FDCs), accounting for 87.4% of psychotropic FDC sales at the time
  ° Recommendations: 9/49 irrational, 7/49 rational, 28/49 requiring further data, 5/49 under consideration by Gupta Committee
  ° Regulatory change: no approvals, 6 formulations allowed de facto approval, 3 FDCs banned

Sales of psychotropic FDCs in 2020 by regulatory status

■ Unapproved formulations accounted for 60.3% of the FDC market
■ There were banned FDCs on the market, including 2 banned as a result of the second initiative

## 4. Discussion

### 4.1. Main findings

There was a proliferation in the market for psychotropic FDCs in India, between 2008 and 2020. The numbers of FDCs and formulations were 17.9% and 41.6% higher in 2020 than in 2008, respectively. Sales of psychotropic FDCs accounted for an increasing proportion of overall psychotropic drug sales (20.1% in 2020 vs 18.4% in 2008). This was driven mainly by growth in the market for antidepressant and benzodiazepine/sedative FDCs.

Unapproved FDCs continued to account for over half of psychotropic FDC sales in 2020, despite the good coverage of the second initiative. The proportions of antipsychotic FDC sales accounted for by unapproved FDCs fluctuated markedly over the study period. This may be due in part to the ban of flupentixol + melitracen in 2014, which had been approved in 1998, but was regarded as unapproved between 2014 and 2017 for the purposes of this study (see Supplemental Material – Appendix A).

Of the 21 FDCs assessed under the initiatives, none went on to be approved and only two were allowed an NOC (de facto approval). Three FDCs went on to be banned and two of these remained on the market in 2020. There were 23 FDCs on the market in 42 formulations in 2020 that were not evaluated by either initiative, including many that would have been eligible.

Both initiatives recommended generation of further data for a number of FDCs. There was no evidence of subsequent CDSCO approval, NOC, or ban for any of these FDCs. The process for evidence generation has been delayed and the requirements diluted, in the case of those assessed under the second initiative (Drugs Controller General (India), 2017b, 2019).

### 4.2. Implications for patients, policy, and research

The use of unapproved or banned psychotropic FDCs could potentially cause significant harm to patients, from both adverse effects and lack of efficacy. There are specific risks associated with the FDCs listed in PharmaTrac. First, many contained combinations of psychotropic drugs. Psychotropic polypharmacy may lead to significant adverse effects such as liver injury and falls (Rothschild, 2021). Second, most of the FDCs contained benzodiazepines or related sedatives, which are associated with misuse and addiction (Guina & Merrill, 2018). Third, some FDCs contained thioridazine, which was withdrawn worldwide in 2005 due to the risk of severe cardiac arrhythmias (Purhonen et al., 2012). Finally, some of the FDCs contained combinations of antipsychotics that are not evidence-based (Galling et al., 2017; Mishra & Gupta, 2022). These risks are compounded by an under-resourced pharmacovigilance system, meaning that there is minimal opportunity to identify harms arising from the use of such FDCs (Sharma, 2021). There are additional economic harms, directly from out of pocket expenditure on the FDCs and indirectly from costs related to the adverse effects of the drugs.

Manufacturers of psychotropic FDCs appear to have subverted the regulatory initiatives, in not submitting FDCs for scrutiny and by taking legal action. It is unclear why the manufacturers were not compelled to submit their FDC data. Their lobbying activities may offer some insights. In a 2014 letter, the president of the Indian Drug Manufacturers' Association argued that India had been ‘a trendsetter in innovative and novel FDCs’ and implied that generation of further data was unnecessary, given that decades of use could provide proof of safety and efficacy (Veerramani, 2014). India's pharmacovigilance system cannot be relied upon to provide the data to substantiate the manufacturers' assertion. It is worth noting that there is limited external (i.e. international) pharmacovigilance data because so few of these FDCs are sold abroad. The manufacturers' views are echoed in the Association's annual report for 2013–14 (Indian Drug Manufacturers' Association, 2014).

### 4.3. Strengths and limitations

This study has several strengths. First, the study used data derived from a large sample of manufacturers and stockists from across India, which should increase confidence in generalisability. Second, the results were compared to those of a previous study that used the same data source (McGettigan et al., 2015), which should increase confidence in reliability. There were a number of limitations. First, there were missing drug and/or form or strength data in PharmaTrac for some FDC formulations. Second, not all of the FDCs mentioned in official documents were listed in PharmaTrac. This could be because they had no measurable sales or because they were sold in an area of India that is not covered by the database. Third, there were discrepancies and missing data in some of the official documents. Finally, FDCs containing psychotropic drugs but primarily marketed for non-psychiatric indications were excluded. It was not possible to ascertain whether these were also used for psychiatric indications. The effect of this would be to underestimate sales and therefore potential harms. There was limited indication information available for FDCs not listed in PharmaTrac (i.e. potentially no longer on the market) and so these were retained in the appendix tables.

### 4.4. Conclusions and recommendations

Psychotropic FDCs are widely marketed in India despite their absence from Indian clinical guidelines, limited evidence of therapeutic benefit, concerns about potential harm, and limited use in other markets. It is clear that further regulatory measures are needed, and that existing measures should be adequately enforced. First, unapproved FDCs should be withdrawn from the market. Second, CDSCO and state regulators should ensure that unapproved and banned FDCs are no longer manufactured. Third, CDSCO should publish the evidence used to justify approvals, NOCs, and bans in full. Fourth, CDSCO should undertake regular audits and market surveillance, to identify unapproved FDCs and intervene. Finally, further research should be carried out to understand the other factors that maintain the market for psychotropic FDCs and to identify other targets for intervention, such as changing public awareness and attitudes. A focus on regulatory measures, without regard for context, is likely to fail.

## Authors' contributions

PBo – conceptualisation, methodology, project administration, data curation (regulatory data), formal analysis (sales data), writing - initial draft, writing – review and editing. AM – conceptualisation, methodology, data curation (sales data), formal analysis (sales data), writing – review and editing. SC – data curation (sales data), formal analysis (sales data), writing – review and editing. PBr - conceptualisation, methodology, writing – review and editing. PR - conceptualisation, formal analysis (regulatory data), writing – review and editing. PM – conceptualisation, methodology, writing – review and editing. HHF – conceptualisation, methodology, writing – review and editing. ANS – conceptualisation, methodology, supervision, writing - review and editing. AMP – conceptualisation, methodology, supervision, writing – review and editing.

## Authors' information

PBo and ANS are consultant psychiatrists. PM is a consultant physician in clinical pharmacology and therapeutics. PBr has expertise in economics. PR has expertise in law. AM, HHF, and AMP have expertise in public health. The team have been researching the use of FDCs in India for over a decade.

## Supplementary Material

Supplemental Material

## Footnotes

1 Note that one FDC was evaluated under both initiatives, which is why the total here is 14 rather than 15.
