# Supplementary material for: Sales and regulatory status of fixed dose combination psychotropic drugs in India: a retrospective longitudinal study
Source: J Pharm Policy Pract. 2024 Aug 6;17(1):2372089. doi: 10.1080/20523211.2024.2372089 (PMC11573336; doi:10.1080/20523211.2024.2372089)
Supplement: Supplemental Material [file JPPP_A_2372089_SM2187.pdf]

Appendix A. The regulatory status of flupentixol + melitracen

Flupentixol + melitracen was approved by CDSCO in 1998. Concerns were subsequently raised about the approval process by the editor of the Monthly Index of Medical Specialities (MIMS) and by a parliamentary committee in 2012 (Department-related Parliamentary Standing Committee on Health and Family Welfare, 2012). The government banned the FDC in June 2013 (Ministry of Health & Family Welfare, 2013a), but this was quashed by the Karnataka High Court in August 2013 (Karnataka High Court, 2013). The FDC was banned again in July 2014 (Ministry of Health & Family Welfare, 2013b) but three years later the court again quashed the ban in July 2017 (Karnataka High Court, 2017). The manufacturer proposed a phase IV trial protocol to the Neurology & Psychiatry Subject Expert Committee which recommended revisions in May 2018 (Central Drugs Standard Control Organization, 2018). In November 2018, the DTAB set up a sub-committee "to examine the issue and submit [a] report for further consideration of the Board" (Drugs Technical Advisory Board, 2018a). In February 2019, the sub-committee requested certain information from the manufacturers which CDSCO sought to operationalise by issuing a public notice (Directorate General of Health Services, 2019). Presumably fearing a ban, the manufacturer challenged the notice in the Karnataka High Court which ordered that no decision should be taken in pursuance of the notice until a phase IV trial had been conducted (Drugs Technical Advisory Board, 2018b). The sub-committee's examination was not completed until April 2021 (Drugs Technical Advisory Board, 2021), and CDSCO approved the protocol in June 2021 with a requirement to complete the trial within two years (Directorate General of Health Services, 2021). In view of the available information, and although this FDC was banned from 2014-2017, it was regarded as not subject to a ban at any point for the purposes of this study. On the other hand, it was regarded as unapproved during the years in which it was technically banned.

References

Central Drugs Standard Control Organization. (2018). *Recommendations of the Subject Expert Committee (Neurology & Psychiatry) made in its 35th meeting held on 11 May 2018, New*

Delhi. Retrieved from https://cdsco.gov.in/opencms/opencms/system/modules/CDSCO.WEB/elements/common_download.jsp?num_id_pk=MzU2 (Accessed: 19 January 2024)

Department-related Parliamentary Standing Committee on Health and Family Welfare. (2012). *Fifty-ninth report on the functioning of the Central Drugs Standard Control Organization (CDSCO)* (Tech. Rep.). Parliament of India. Retrieved from https://www.casemindia.org/wp-content/uploads/2020/05/59th-Report-of-the-Parliamentary-Standing-Committee-on-Health-on-the-Functioning-of-the-CDSCO-2012.pdf (Accessed: 21 February 2024)

Directorate General of Health Services. (2019). *Public Notice. Consideration of the orders of High Court of Karnataka dated 14.08.2013 & 24.07.2017 to examine the issue of safety and efficacy of fixed dose combination of Flupenthixol + Melitracen for human use in light of notifications GSR 377(E) dated 18.6.2013 & GSR 498(E) dated 11.07.2014 and to provide hearing to the petitioners/manufacturers.* Retrieved from https://cdsco.gov.in/opencms/resources/UploadCDSCOWeb/2018/UploadPublic_NoticesFiles/Publicnotiec13feb19.pdf (Accessed: 19 January 2024)

Directorate General of Health Services. (2021). *Letter to M/s Mankind Pharma. Permission to conduct phase IV clinical trial with the FDC of flupentixol 0.5 mg + melitracen 10 mg tablets (vide protocol no. CRB/CT-001/2018, version no. 3.1, dated 01.12.2020). ct-06-63/2021.* Retrieved from https://cdsco.gov.in/opencms/resources/UploadCDSCOWeb/2018/UploadCTApprovals/4.%20Flupentixol%20+%20Melitracen%20tablets%20(Phase%20IV).pdf (Accessed: 19 January 2024)

Drugs Technical Advisory Board. (2018a). *Minutes of the 81st meeting of Drugs Technical Advisory Board.* Retrieved from https://cdsco.gov.in/opencms/opencms/system/modules/CDSCO.WEB/elements/common_download.jsp?num_id_pk=NTY2 (Accessed: 21 August 2022)

Drugs Technical Advisory Board. (2018b). *Minutes of the 83rd meeting, 11 June 2019, Additional Agenda item no. S-1.* Retrieved from https://cdsco.gov.in/opencms/opencms/system/modules/CDSCO.WEB/elements/common_download.jsp?num_id_pk=OTM5 (Accessed: 19 January 2024)

Drugs Technical Advisory Board. (2021). *Minutes of the 86th meeting of Drugs Technical Advisory Board.* Retrieved from https://cdsco.gov.in/opencms/opencms/system/modules/CDSCO.WEB/elements/common_download.jsp?num_id_pk=MTM3NA== (Accessed: 23 June 2022)

Karnataka High Court. (2013). *Lundbeck India Private Limited vs Union of India, W.P. No.28354/2013.* Retrieved from https://indiankanoon.org/doc/166489410/ (Accessed: 19 January 2024)

Karnataka High Court. (2017). *Mankind Pharma Limited vs Union of India, W.P. Nos. 318-322/2014.* Retrieved from https://indiankanoon.org/doc/150135244/ (Accessed: 19 January 2024)

Ministry of Health & Family Welfare. (2013a). *The Gazette of India. Extraordinary, part II, section 3, sub-section (i). Notification GSR 377(E).* Retrieved from https://cdsco.gov.in/opencms/opencms/system/modules/CDSCO.WEB/elements/download_file_division.jsp?num_id=MTMyMg== (Accessed: 19 January 2024)

Ministry of Health & Family Welfare. (2013b). *The Gazette of India. Extraordinary, part II, section 3, sub-section (i). Notification GSR 498(E).* Retrieved from https://cdsco.gov.in/opencms/opencms/system/modules/CDSCO.WEB/elements/download_file_division.jsp?num_id=MTQwNQ=== (Accessed: 19 January 2024)

Appendix B. The regulatory status of imipramine + diazepam.

There is no record of CDSCO approval for imipramine + diazepam, which was first manufactured prior to 21 September 1988 (a "pre-1988" FDC). The Kokate Committee categorised the two marketed formulations of this FDC as irrational. The government banned the FDC in March 2016 (Ministry of Health & Family Welfare, 2016), but this was stayed by the High Court of Delhi (Delhi High Court, 2016). The Supreme Court set aside the ban in December 2017 on the ground that pre-1988 FDCs "were never meant to be referred to the Kokate Committee" (Supreme Court of India, 2017). Such FDCs were originally within the scope of the second initiative when it was launched in January 2013, but they were subsequently excluded. Although the Supreme Court did not remit this FDC to the DTAB, it was evaluated by the Kshirsagar Committee which recommended prohibition. No evidence of any subsequent ban has been found. A further committee was set up by the government in 2021, chaired by Prof MS Bhatia, to examine 19 pre-1988 FDCs, including imipramine + diazepam (Directorate General of Health Services, 2021). The research team were unable to locate published reports corresponding to the work of this committee, though the report was examined by the DTAB in September 2022 (Drugs Technical Advisory Board, 2022). In view of the available information, this FDC was regarded as not subject to a ban for the purposes of this study.

References

Delhi High Court. (2016). *Pfizer Limited vs Union of India, W.P.(C) No. 2212/2016.* Retrieved from `https://indiankanoon.org/doc/16479147/` (Accessed: 19 January 2024)

Directorate General of Health Services. (2021). *Notice. Evaluation of certain pre 1988 permitted fixed dose combinations (FDCs) de novo for manufacture for sale in the country without due approval from central licensing authority. 26 July 2021. File No. 4-01/2013-DC (Misc. 13 PSC Part II).* Retrieved from `https://cdsco.gov.in/opencms/opencms/system/modules/CDSCO.WEB/elements/download_file_division.jsp?num_id=NzQ1Nw==` (Accessed: 19 January 2024)

Drugs Technical Advisory Board. (2022). *Minutes of the 88th meeting held on 26 Septem-*

ber 2022. Retrieved from https://cdsco.gov.in/opencms/opencms/system/modules/CDSCO.WEB/elements/common_download.jsp?num_id_pk=MTc4Mg== (Accessed: 19 January 2024)

Ministry of Health & Family Welfare. (2016). *The Gazette of India. Extraordinary, part II, section 3, sub-section (ii). Notifications SO 705(E) to SO 1048(E).* Retrieved from https://cdsco.gov.in/opencms/opencms/system/modules/CDSCO.WEB/elements/download_file_division.jsp?num_id=MTA2MQ==

Supreme Court of India. (2017). *Union of India vs Pfizer Limited, Civil Appeal No. 22972/2017.* Retrieved from https://indiankanoon.org/doc/179615704/ (Accessed: 19 January 2024)

Appendix C. Supplementary tables

Table C1. Psychotropic FDCs on the market between 2008 and 2020

Antipsychotic	
	Chlorpromazine + trifluoperazine + trihexyphenidyl, chlorpromazine + trihexyphenidyl
	Flupentixol + escitalopram, flupentixol + melitracen
	Haloperidol + trihexyphenidyl
	Olanzapine + fluoxetine
	Risperidone + trihexyphenidyl
	Thioridazine + trihexyphenidyl, thioridazine + trifluoperazine + trihexyphenidyl
	Trifluoperazine + chlordiazepoxide, trifluoperazine + chlordiazepoxide + trihexyphenidyl, trifluoperazine + imipramine + chlordiazepoxide + trihexyphenidyl, trifluoperazine + trihexyphenidyl
Antidepressant	
	Amitriptyline + chlordiazepoxide
	Desvenlafaxine + clonazepam
	Escitalopram + clonazepam, escitalopram + etizolam, escitalopram + L-methylfolate
	Fluoxetine + alprazolam
	Imipramine + alprazolam, imipramine + chlordiazepoxide, imipramine + diazepam
	Paroxetine + clonazepam
	Sertraline + alprazolam
Benzodiazepine/sedative	
	Alprazolam + melatonin, alprazolam + propranolol
	Clonazepam + propranolol
	Diazepam + propranolol
	Etizolam + propranolol
	Melatonin + zolpidem

FDC = fixed dose combination.

Table C2. Psychotropic FDCs with CDSCO approval

FDC	Formulation	Year of approval	Year first listed in PharmaTrac
Chlorpromazine + trifluoperazine + trihexyphenidyl	50mg/5mg/2mg T	1979	2008*
Escitalopram + clonazepam	5mg/0.5mg T, 10mg/0.5mg T	2004, 2007	2008*
Flupentixol + melitracen	0.5mg/10mg T	1998†	2008*
Olanzapine + fluoxetine	5mg/20mg T, 10mg/20mg T	2003	2008*
Paroxetine + clonazepam	12.5mg/0.5mg T CR, 25mg/0.5mg T CR	2010	2013
Sertraline + alprazolam	25mg/0.25mg T, 25mg/0.5mg T, 50mg/0.25mg T, 50mg/0.5mg T	2006	2008*

*Note that it was not possible to ascertain whether these FDCs were on the market prior to 2008 due to the limitations of the PharmaTrac data. † This FDC was regarded as unapproved from 2014 to 2017, for the purposes of this study (see Appendix A). CR = controlled release, FDC = fixed dose combination, T = tablet.

Table C3. Psychotropic FDCs considered by the Gupta Committee that were listed in PharmaTrac, with committee recommendations.

FDC	Committee recommendation
Alprazolam + melatonin	Further trial data
Alprazolam + propranolol	Further trial data
Diazepam + propranolol	Further trial data

FDC = fixed dose combination.

Table C4. Additional FDCs containing psychotropic drugs considered by the Gupta Committee that were not listed in PharmaTrac, with committee recommendations.

FDC	Committee recommendation
Alprazolam + paracetamol	Already banned
Chlordiazepoxide + clidinium + dicyclomine + paracetamol	Insufficient data
Chlordiazepoxide + dextropropoxyphene + dicyclomine + paracetamol	Irrational
Chlordiazepoxide + dicyclomine + paracetamol	Already banned
Diazepam + dicyclomine + dipyrone	Already banned
Diazepam + diphenhydramine + dipyrone	Already banned
Diazepam + dipyrone	Already banned
Diazepam + dipyrone + paracetamol	Already banned
Diazepam + dipyrone + propylene glycol	Already banned

FDC = fixed dose combination.

Table C5. Psychotropic FDC formulations considered by the Kokate Committee that were listed in PharmaTrac, by committee recommendation.

FDC	Formulation
Under consideration by Gupta Committee	
Alprazolam + melatonin	0.25mg/3mg T, 0.5mg/3mg T
Alprazolam + propranolol	0.125mg/20mg T, 0.25mg/10mg T, 0.25mg/20mg T
Irrational	
Chlorpromazine + trihexyphenidyl	100mg/2mg T, 200mg/2mg T
Etizolam + propranolol	0.25mg/20mg T, 0.5mg/20mg T, 0.5mg/40mg T
Flupentixol + escitalopram	0.5mg/10mg T
Flupentixol + melitracen	0.5mg/10mg T
Imipramine + diazepam	25mg/2mg T, 25mg/5mg T
Rational	
Escitalopram + clonazepam	5mg/0.25mg T, 10mg/0.25mg T, 10mg/0.5mg T, 20mg/0.5mg T
Risperidone + trihexyphenidyl	2mg/2mg T, 3mg/2mg T, 4mg/2mg T
Requiring further data	
Active post-marketing surveillance	
Amitriptyline + chlordiazepoxide	12.5mg/5mg T, 25mg/5mg T, 25mg/10mg T and T XL
Clonazepam + propranolol	0.25mg/10mg T, 0.25mg/20mg T, 0.5mg/10mg T, 0.5mg/20mg T
Desvenlafaxine + clonazepam	50mg/0.5mg T
Diazepam + propranolol	2.5mg/20mg T
Escitalopram + etizolam	5mg/0.5mg T, 10mg/0.5mg T, 10mg/1mg T
Fluoxetine + alprazolam	20mg/0.25mg T, 20mg/0.5mg T ‡
Sertraline + alprazolam	25mg/0.5mg T, 50mg/0.25mg T, 50mg/0.5mg T ‡
Trifluoperazine + chlordiazepoxide	1mg/10mg T, 2mg/10mg T
Trifluoperazine + chlordiazepoxide + trihexyphenidyl	1mg/10mg/2mg T†
Trifluoperazine + trihexyphenidyl	2.5mg/1mg T, 5mg/2mg T
Phase IV trial	
Melatonin + zolpidem	3mg/5mg T, 3mg/10mg T
Bioequivalence study	
Paroxetine + clonazepam	12.5mg/0.5mg T and T CR, 25mg/0.5mg T and T CR

† There were no measurable sales for this formulation in 2014. ‡ Recommendations regarding the type of further data were missing for at least one formulation. CR = controlled release, FDC = fixed dose combination, T = tablet, XL = extended release.

Table C6. Additional FDC formulations containing psychotropic drugs considered by the Kokate Committee that were either not listed in PharmaTrac or listed but with missing data, by committee recommendation and with requirements for further data.

FDC	Formulation
Further expert consideration	
Chlordiazepoxide + clidinium + dicyclomine + rabeprazole	5mg/2.5mg/10mg/10mg C ER
Irrational	
Diazepam + dried aluminum hydroxide gel + propantheline	2mg/100mg/15mg C
Escitalopram + calcium L-5 methyltetrahydrofolate*	10mg/7.5mg T
Trifluoperazine + imipramine + chlordiazepoxide + trihexyphenidyl†	1.5mg/25mg/10mg/0.5mg T
Rational	
Chlordiazepoxide + clidinium + drotaverine	5mg/2.5mg/80mg T
Requiring further data	
Phase IV trial	
Dosulepin + methylcobalamin + pregabalin	75mg/75mg C, 150mg/75mg C‡
Unclear	
Dosulepin + pregabalin	75mg/75mg/1500mcg C, 150mg/75mg/1500mcg C‡

* This was considered to be different from the FDC escitalopram + L-methylfolate, which was listed in PharmaTrac because calcium L-5 methyltetrahydrofolate and L-methylfolate are different chemical compounds. † This FDC appears in PharmaTrac but without any strength data. ‡ The strengths for these two FDCs appear to have been interchanged in error in the committee's report. C = capsule, ER = extended release, FDC = fixed dose combination, T = tablet.

Table C7. Psychotropic FDC formulations that were allowed a no objection certificate (NOC), following evaluation by the Kokate Committee.

FDC	Formulation	Year of NOC
Escitalopram + clonazepam	5mg/0.25mg T, 10mg/0.25mg T, 20mg/0.5mg T	2015
Risperidone + trihexyphenidyl	2mg/2mg T, 3mg/2mg T, 4mg/2mg T	2016

FDC = fixed dose combination, NOC = no objection certificate, T = tablet.

Table C8. Psychotropic FDCs considered by the Gupta Committee that were listed in PharmaTrac, with requirements for further data.

FDC	Data required
Alprazolam + melatonin	Active post-marketing surveillance
Alprazolam + propranolol	Phase IV trial
Diazepam + propranolol	Phase IV trial

FDC = fixed dose combination.

Table C9. Sales volumes and regulatory status for the ten psychotropic FDCs with the highest sales in 2020.

FDC	Category	Sales (millions SU)	Regulatory status
Escitalopram + clonazepam	Antidepressant	424.9	Approved*
Amitriptyline + chlordiazepoxide	Antidepressant	245.0	Unapproved
Risperidone + trihexyphenidyl	Antipsychotic	127.3	NOC*
Flupentixol + melitracen	Antipsychotic	107.3	Approved*†
Trifluoperazine + trihexyphenidyl	Antipsychotic	98.8	Unapproved
Clonazepam + propranolol	Benzodiazepine/sedative	69.7	Unapproved
Escitalopram + etizolam	Antidepressant	58.0	Unapproved
Alprazolam + propranolol	Benzodiazepine/sedative	55.9	Unapproved
Etizolam + propranolol	Benzodiazepine/sedative	54.1	Unapproved
Paroxetine + clonazepam	Antidepressant	40.1	Approved*

* At least one formulation has CDSCO approval or NOC, though a proportion of sales may correspond to formulations with neither. † This FDC has a complicated regulatory history (see Appendix A). CDSCO = Central Drugs Standard Control Organization, FDC = fixed dose combination, NOC = no objection certificate, SU = standard units.
